# Re-engaging an inactive cohort of young adults: evaluating recruitment for the Kidskin Young Adult Myopia Study

**DOI:** 10.1186/s12874-020-00996-y

**Published:** 2020-05-24

**Authors:** Gareth Lingham, David A. Mackey, Nicola Seed, Lisa Ryan, Elizabeth Milne, Robyn M. Lucas, Maria Franchina, Samantha Sze-Yee Lee, Seyhan Yazar

**Affiliations:** 1 Centre for Ophthalmology and Visual Sciences, Lions Eye Institute, University of Western Australia, 2 Verdun St, Nedlands WA, Perth, 6009 Australia; 2 Telethon Kids Institute, University of Western Australia, Perth, Australia; 3 National Centre for Epidemiology and Population Health, Research School of Population Health, Australian National University, Canberra, Australia; 4 Garvan Institute of Medical Research, Sydney, Australia

**Keywords:** Retention, Recruitment, Cohort, Young adult

## Abstract

**Background:**

Recent changes in communication technologies, including increased reliance on mobile phones and the internet, may present challenges and/or opportunities to re-engaging inactive study cohorts. We evaluate our ability to recruit participants for the Kidskin Young Adult Myopia Study (KYAMS), a follow-up of the Kidskin Study.

**Methods:**

KYAMS participants were recruited from the Kidskin Study, a sun exposure-intervention study for 5–6 year-olds running from 1995 to 1999 with most recent follow-up in 2005. From 2015 to 2019, the KYAMS used mail-outs, phone calls and social media to contact Kidskin Study participants. Multivariable logistic regression was used to identify variables associated with successful contact of a Kidskin Study participant or family member and KYAMS participation.

**Results:**

Of 1695 eligible participants, 599 (35.5%) participants (or a family member) were contacted and 303 (17.9%) participated in the KYAMS. KYAMS participation was more likely in those who participated in the 2005 follow-up (odds ratio [OR] = 5.09, 95% confidence interval [CI]: 3.67–7.06) and had a mobile phone number on record (OR = 2.25, CI: 1.57–3.23). Of those contacted, participants who were the first point of contact (OR = 4.84, CI: 2.89–8.10) and who were contacted by letter in the first (OR = 6.53, CI: 3.35–12.75) or second (OR = 5.77, CI: 2.85–11.67) round were more likely to participate in the KYAMS, compared to contact by landline phone.

**Conclusions:**

We recruited approximately one-fifth of Kidskin Study participants for the KYAMS. Participants were more likely to participate in the KYAMS if they were contacted directly, rather than through a family member, and if they were contacted by invitation letter.

**Trial registration:**

ACTRN12617000812392.

## Background

Current lifestyle and environmental factors can affect future health. For example, childhood or adolescent obesity is associated with higher risk of mortality in adulthood [1] and exposure to ultraviolet radiation during childhood has been linked to increased risk of melanoma in adulthood [2, 3]. To fully investigate the long-term effects of childhood lifestyle and environment on health, it may be necessary to conduct long-term, prospective, cohort studies; however, these studies are costly to run and maintain. A cheaper and quicker alternative to a prospective cohort study may be to leverage off a previously established cohort and re-engage or recruit its participants for a follow-up. For example, we conducted the Kidskin Young Adult Myopia Study (KYAMS), a follow-up of the Kidskin Study. The Kidskin Study was initially established to investigate the effects of sun exposure during childhood (age 5–12 years) on development of melanocytic naevi, a marker of melanoma risk. The KYAMS focussed on eye health in young adulthood (25–30 years of age) but used data on sun exposure collected during the Kidskin Study to investigate the long-term effects of childhood sun exposure on myopia (near-sightedness) [4].

Re-engaging a cohort that has not been actively followed-up for some years, or even decades, is challenging. Participants may change address or phone number or relocate interstate or internationally. The means by which we communicate have changed rapidly over the last two decades. Mobile phones and communication via social media and other online platforms has become more common, whereas communication by post or landline telephone has declined. These changes could present additional challenges and/or opportunities for re-initiating contact with cohort members. For example, the reduction in use of landline phones may mean that phone numbers collected during a previous study have become obsolete. On the other hand, the rise of social media may facilitate contact with study participants. Some studies, conducted in the mid-2000s, have evaluated their success in retaining cohort study members after long periods of inactivity (6–20 years) [5, 6]. However, there is little empirical evidence on how best to re-engage a cohort after a loss of contact over a long period in the context of recent changes in communication methods. We aimed to retrospectively evaluate our ability to recruit Kidskin Study participants for the KYAMS.

## Methods

### The Kidskin Study

The Kidskin Study was a non-randomised controlled trial that aimed to reduce sun exposure in children through a specially designed educational intervention. The Kidskin Study began in 1995 and recruited children attending their first year of school (aged 5 to 6 years) at one of 33 participating schools in Perth, Western Australia. Schools were assigned to a control group, a moderate-intensity intervention (moderate) group, or a high-intensity intervention (high) group [7]. Children received the intervention between 1995 and 2001 and were invited to participate in assessments for the Kidskin Study in 1995, 1997 (8–9 years), 1999 (10–11 years) and 2001 (12–13 years) [8, 9]. At the Kidskin Study baseline, parents or caregivers (hereafter referred to as parents) completed questionnaires that included questions on child’s sex, ancestry, tendency to sunburn or suntan after sun exposure and educational attainment of parents. Contact details were updated at each follow-up.

**Fig. 1:**
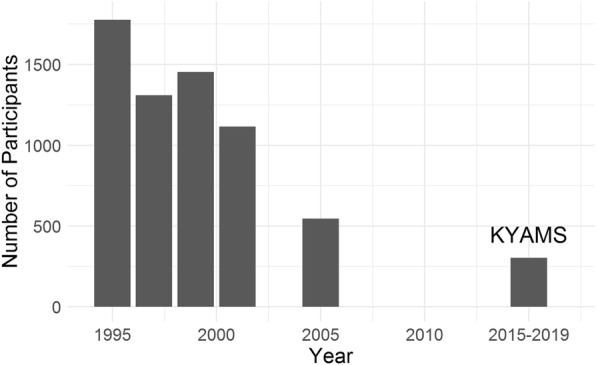
Bar chart showing decline in Kidskin Study participation over time. Follow-ups of the Kidskin Study were conducted in 1995, 1997, 1999, 2001 and 2005 and the KYAMS ran between 2015 and 2019

A further follow-up of Kidskin Study participants was conducted in 2005 (age 16–17 years) in which participants were invited by mail to provide saliva samples for genetic analysis. At the 2005 follow-up, participants were asked to provide a landline and mobile telephone number (where able). Active participation in the Kidskin Study gradually declined over time, with 1776 participants enrolled at baseline in 1995 and 547 (30.8%) consenting to the 2005 follow-up (Fig. 1).

### The KYAMS

The KYAMS was a follow-up of the Kidskin Study conducted between May 2015 and March 2019. The KYAMS investigated the effect of childhood sun exposure on myopia in young adulthood. Participation in the study involved a 2- to 3-h eye examination at the Lions Eye Institute in Perth, Western Australia [4]. All participants of the Kidskin Study who had not withdrawn consent were eligible to participate in the KYAMS. Ethics approval to conduct the KYAMS was obtained from the University of Western Australia Human Research Ethics Committee.

### KYAMS recruitment methods

**Fig. 2:**
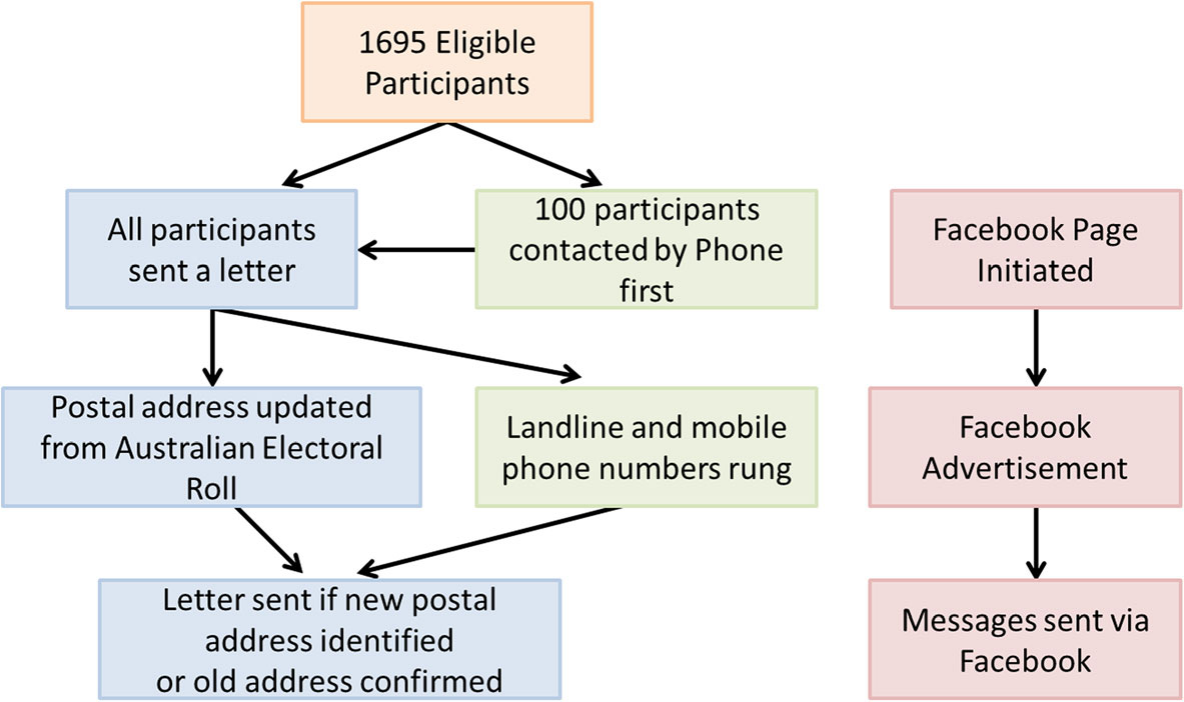
Flow chart showing recruitment methods for KYAMS. If direct contact was made with the participants, we did not attempt to make contact using subsequent contact methods

Methods for KYAMS recruitment are outlined in Fig. 2. We used contact data collected at the participant’s most recent Kidskin Study follow-up. We did not attempt to make contact by any method with individuals for whom we had no telephone number or postal address.

A sample of 100 participants, randomly selected from those who participated in the 2005 follow-up using the ‘sample’ function in R (R Foundation for Statistical Computing, Vienna, Austria), were initially contacted by phone. Another random sample of 100 participants of the 2005 follow-up were contacted by letter, which included a reply-paid envelope and a short form for participants to update their contact information and indicate whether they would like to participate. A higher participation rate was observed in the group first contacted by letter (25 vs 17 participants). Consequently, all remaining participants were sent a letter as the first point of contact. After the first round of invitation letters, we used the Australian Electoral Roll to obtain updated postal addresses where able, while also confirming old postal addresses from which we had no response. Registration on the Australian Electoral Roll is compulsory for Australian citizens over 18 years of age and over 95% of those estimated to be eligible for the Electoral Roll are enrolled [10]. The Roll contains only names and addresses; therefore, we could rarely differentiate between multiple records of people with the same first and last name.

All telephone numbers (both mobile and landline) were rung at least once. Many were rung multiple times, but these data were not recorded. Telephones were often answered by parents and occasionally siblings. When this happened, either the family member provided contact details for the participant or the researcher provided details of the study and how to contact the study team for the family member to pass on to the participant, depending on the preference of the family member.

A Facebook page was set up at the initiation of the KYAMS and participants were encouraged to follow the page and share it with their friends. An advertisement was placed on Facebook in late 2017 to early 2018. The advertisement was targeted to friends of participants who followed the study page. Those who clicked on the advertisement were directed to a form where they received more information about the study and could provide their contact details if interested. Finally, we searched first and last names to identify Kidskin Study participants on Facebook and sent a message inviting them to participate in the study. As we were not ‘Friends’ with participants on Facebook, messages were not sent directly to participants but rather ended up in a “message request” folder, which required participants to accept the request before viewing the message. Once a Kidskin Study participant had been directly contacted (i.e. not via family or friends), we did not attempt to contact them by other means. For example, Facebook messages that were sent later in the recruitment period (e.g. 2018) were not sent to those already contacted by phone or mail. Throughout the KYAMS, participants were also encouraged to tell their school friends about the study.

Participants who had been contacted using any of the approaches were classified as either: participated, interested in participating (but did not participate), not living in Perth (and therefore unable to participate), family member to pass on details (but no further contact made), or contacted but not interested. Contact information including details of any successful contact were recorded in a spreadsheet. Data for this analysis were retrospectively extracted from the spreadsheet.

### Statistical analysis

Parental education was collapsed into tertiary vs non-tertiary qualification. Ancestry was categorised into European (British, Northern Europe, Southern Europe ancestry) or non-European. Demographic data based on Kidskin Study baseline (1995) questionnaire data were compared in participants of the Kidskin Study baseline and KYAMS participants using Pearson’s Chi square test. The significance level was set at 0.05.

The primary outcome in the analysis was participation in the KYAMS (yes/no, referred to as KYAMS participation). The secondary outcome was contact made with the participant or a family member (yes/no, referred to as KYAMS contact). We calculated contact attempts in different ways for each method. For letters, contact attempts were the number of letters sent. For phones, each available phone number (mobile or landline) was considered one contact attempt. For Facebook, one Facebook message sent was considered one contact attempt and one click on the advertisement was one contact attempt. We could not estimate contact attempts for the Facebook page or word of mouth. The first point of contact was the person (participant, sibling or parent) who was first contacted by the study team. Due to low numbers of siblings (*n* = 13) being the first point of contact, we collapsed the parent and sibling groups together. A participant’s phone was classed as “disconnected” if all telephone numbers were disconnected.

Logistic regression was used to analyse the association between demographics and available contact data at the beginning of the KYAMS and participation in the KYAMS (primary outcome) or contact being made with the participant or a family member (secondary outcome). For those who were contacted, we additionally investigated whether the first point of contact (participant vs family member), or a particular contact method, was more likely to result in KYAMS participation. All statistical analyses were performed in R version 3.5.1 (R Foundation for Statistical Computing, Vienna, Austria).

## Results

Of the 1776 participants who consented to the Kidskin Study in 1995, 1728 (97.3%) had not withdrawn consent. Of these, 33 (1.9%) did not have a phone number or home address and were excluded from this analysis. Of the remaining 1695 participants, 1694 had a home address, 1626 (95.9%) had a landline phone number and 415 (24.5%) had a mobile phone number prior to the KYAMS. A large proportion of those with a mobile phone number had participated in the 2005 follow-up (*n* = 388, 93.5%). Participants’ phones were disconnected in 415 (33.9%) of those with only a landline number and 77 (18.6%) of those with a mobile number, with or without a landline number. From the Australian Electoral Roll we confirmed the address of 147 (8.7%) participants and identified a new address for 697 (41.1%) participants.

Between May 2015 and March 2019, 599 (35.3%) Kidskin Study participants or a family member were contacted and 303 (17.9%) individuals from the original cohort (50.6% of those contacted) participated in the KYAMS. Approximately two-thirds (*n* = 205, 67.7%) of KYAMS participants had participated in the 2005 follow-up. The mean age of KYAMS participants was 27.5 years (range: 25.2–30.0 years), and compared to baseline characteristics in the Kidskin Study, KYAMS participants were more likely to be female, be in an intervention group and have a parent with a tertiary education (Table 1). Furthermore, after adjusting for the effect of intervention group, KYAMS participants had lower sun exposure (measured in midday hour equivalents) over the previous summer holidays as reported by parents in 1997 (beta = − 6.5, 95% confidence interval [CI]: − 0.6, − 12.3), 1999 (beta = − 6.9, 95% CI: − 0.7, − 13.1) and 2001 (− 8.7, 95% CI: − 0.5, − 17.0), compared to those who did not participate in the KYAMS. There was no significant difference in parent-reported sun exposure over the summer holidays at baseline (1995).

**Table 1:** Demographics of participants at the Kidskin Study baseline and in the KYAMS

Demographics	Kidskin Study (*n* = 1695)	KYAMS (*n* = 303)	p^a^
Sex			< 0.001
Male	854 (51.5%)	116 (38.5%)	
Female	803 (48.5%)	185 (61.5%)	
Intervention Group			0.002
Control	753 (45.4%)	101 (33.6%)	
Moderate	397 (23.9%)	90 (29.9%)	
High	510 (30.7%)	110 (36.5%)	
Highest Parental Education			0.005
Non-tertiary	1019 (62.2%)	160 (53.5%)	
Tertiary	618 (37.8%)	139 (46.5%)	
European ancestry			0.07
No	181 (10.9%)	22 (7.3%)	
Yes	1479 (89.1%)	279 (92.7%)	
Sunburn after sun exposure^b^			0.94
Get severe sunburn with blistering	198 (12.0%)	36 (12.0%)	
Have painful sunburn	695 (42.2%)	132 (44.0%)	
Get mildly burnt	661 (40.1%)	115 (38.3%)	
Not get sunburnt at all	93 (5.6%)	17 (5.7%)	
Tanning after sun exposure^c^			0.07
Very tanned	584 (35.4%)	84 (28.0%)	
Moderately tanned	677 (41.1%)	144 (48.0%)	
Lightly tanned	339 (20.6%)	64 (21.3%)	
No suntan at all	48 (2.9%)	8 (2.7%)	

^a^ Pearson Chi Square test

^b^ Parent-reported sunburn after spending 30 min in the sun in the middle of the day for the first time in summer without wearing sunscreen

^c^ Parent-reported sun tanning at the end of summer after spending short periods of time in the sun every day over summer without sunscreen

**Fig. 3:**
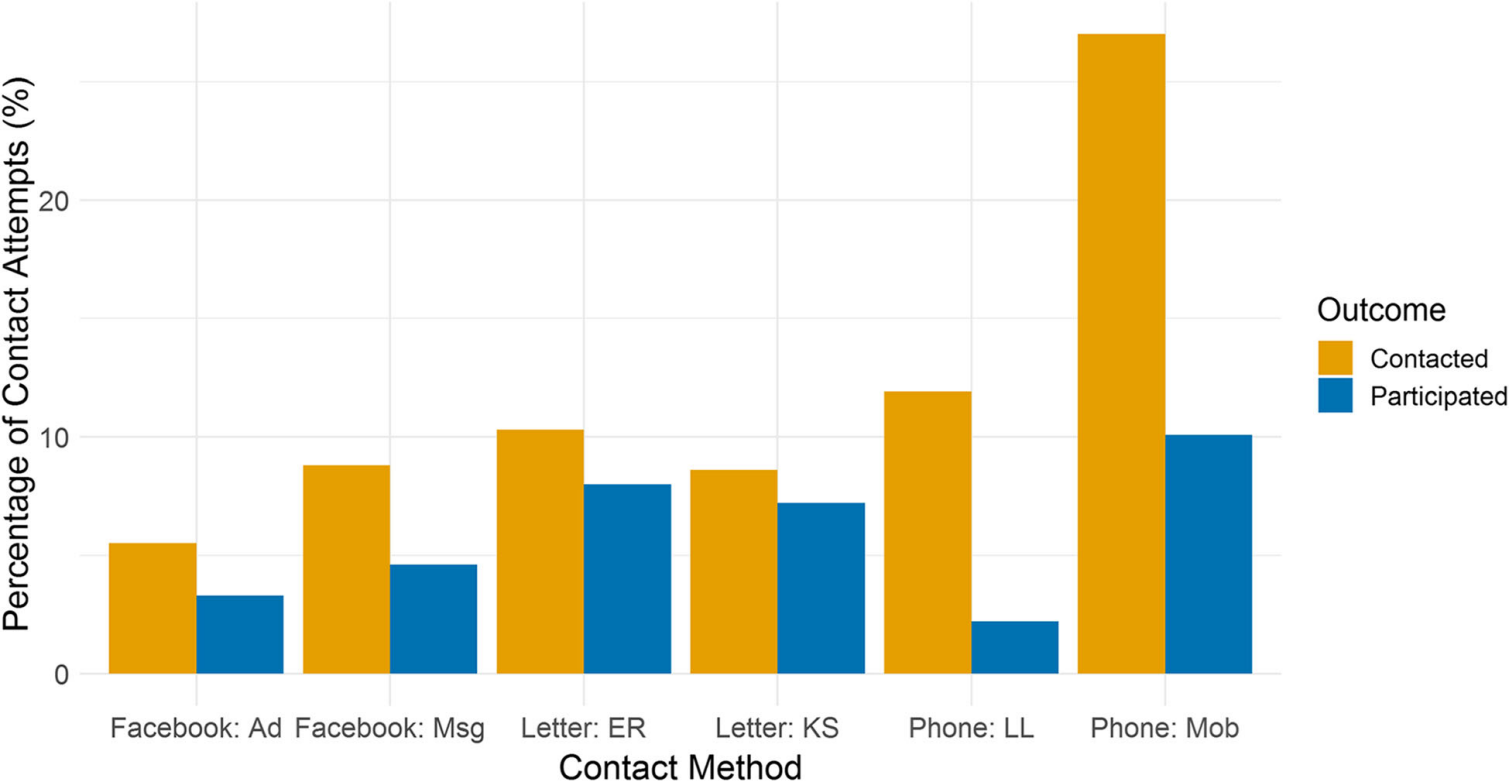
Percentage of contact attempts resulting in either contact with the participant or their family member (Contacted) or KYAMS participation (Participated) for each contact method. Number of contact attempts: Facebook: Ad (Advertisement) = 273, Facebook: Msq (Message) = 364, Letter: ER (Electoral Roll address) = 884, Letter: KS (Previous Kidskin Study address) = 1694, Phone: LL (Landline) = 1626, Phone: Mob (Mobile) = 415. Figure does not include 6 participants who were contacted by word of mouth, 3 of whom participated in the KYAMS, and 3 participants who contacted us via the Facebook study page, all of whom participated

A total of 5526 contact attempts were made including 2578 (46.7%) letters sent, 2041 (36.9%) phone numbers called, 364 (6.6%) Facebook messages sent and 273 (4.9%) Facebook advertisement clicks. Figure 3 shows the breakdown of successful instances of KYAMS contact or participation. Of the 5526 contact attempts, 599 were successful. Most instances of contact were made by phone (*n* = 302, 50.4%), followed by letter (*n* = 236, 39.4%), Facebook (*n* = 50, 8.3%) and word of mouth (*n* = 6, 1.0%). Of the 303 participants who consented to the KYAMS, 193 (63.7%) were contacted by letter, 78 (25.7%) were contacted by phone, 29 (9.6%) by Facebook and 3 (1.0%) by word of mouth. When the participant was the first point of contact, contact resulted in KYAMS participation in 257 of 357 (72.0%) instances compared to just 46 of 242 (19.0%) instances where the parent or sibling was the first point of contact.

Of the 296 participants (or family members) who were contacted but did not participate, 45 (15.2%) participants initially expressed an interest in the KYAMS but were ultimately unable to organise an appointment, 62 (21.0%) did not reside in Perth and were unable to participate on return trips, 82 (27.7%) declined to participate and, in 102 (34.5%) instances, details of the study and how to contact the study team were left with a family member.

The results of the multivariable logistic regression models for both the primary and secondary outcomes are shown in Table 2. Participation in the 2005 follow-up was associated with 5-fold and 4-fold higher odds of KYAMS contact and participation, respectively. Females and those in the intervention groups were also more likely to be contacted and participate in the KYAMS. Those with a mobile or landline number had approximately 2-fold higher odds of KYAMS contact; however, only having a landline phone number was significantly associated with increased likelihood of KYAMS participation.

**Table 2:** Multivariable analysis of factors associated with contact or participation

Multivariable model	Contacted (*n* = 1656)	Participated (*n* = 1618)	Participation after contact (*n* = 599)
	Odds ratio (95% CI)	Odds ratio (95% CI)	Odds ratio (95% CI)
Sex			
Male	Reference	Reference	Reference
Female	1.51 (1.20, 1.91)	1.83 (1.39, 2.42)	1.31 (0.87, 1.97)
Intervention group			
Control	Reference	Reference	Reference
Moderate	1.43 (1.09, 1.88)	1.68 (1.22, 2.32)	1.25 (0.76, 2.04)
High	1.60 (1.20, 2.14)	1.68 (1.19, 2.36)	1.35 (0.82, 2.22)
Participant 2005 follow-up			
No	Reference	Reference	Reference
Yes	5.09 (3.67, 7.06)	4.22 (2.85, 6.25)	1.87 (1.15, 3.04)
Mobile number			
No	Reference	Reference	NA
Yes	2.25 (1.57, 3.23)	1.43 (0.96, 2.11)	NA
Landline number			
No	Reference	Reference	NA
Yes	1.96 (1.04, 3.67)	2.45 (1.02, 5.86)	NA
Telephone/s disconnected			
No	NA	Reference	NA
Yes	NA	0.44 (0.31, 0.64)	NA
First point of contact			
Parent/Sibling	NA	NA	Reference
`Participant	NA	NA	4.84 (2.89, 8.10)
Contact method			
Phone: Landline	NA	NA	Reference
Phone: Mobile	NA	NA	1.54 (0.83, 2.84)
Letter: Old address	NA	NA	6.53 (3.35, 12.74)
Letter: Electoral roll address	NA	NA	5.77 (2.85, 11.67)
Facebook: Message	NA	NA	2.01 (0.81, 4.98)
Facebook: Advertisement/Page^a^	NA	NA	2.72 (0.84, 8.81)
Word of mouth	NA	NA	2.06 (0.37, 11.59)

^a^All participants contacted through the Facebook page participated, therefore this group was combined with those contacted through the Facebook advertisement

When we restricted the analysis to those who had been contacted, we found that when the participant was the first point of contact (using any contact method), the likelihood of participating in the KYAMS was nearly 5-times higher. Being contacted by letter – either using the Kidskin Study address or the Electoral Roll address – was associated with approximately 6-times higher odds of participating in the KYAMS when compared to being contacted by landline telephone, which had the least success. In this restricted analysis, participating in the 2005 follow-up was still associated with higher likelihood of participating in the KYAMS, although the effect had substantially lessened, and sex and intervention group were no longer significantly associated with KYAMS participation.

### Power

Assuming equal numbers of participants in the exposure groups, we needed a sample size of 812 participants to have 80% power to detect a difference in participation rates of 8% (based on the pilot study). Thus our study was generally well-powered to detect differences. Even when analyses were restricted to the 599 participants who had been contacted, we had 67% power to detect an 8% difference in participation rates between groups.

## Discussion

We retrospectively evaluated our success in re-engaging the Kidskin Study cohort after at least 10 years of inactivity. The number of KYAMS participants (*n* = 303) was consistent with the decline in Kidskin Study participation over time (Fig. 1). Our retention rate (17.9%) was lower than for other long-term follow-ups of similarly aged participants (43 to 80%) [11–13]. Many of these studies were designed for long-term follow-up, thus participants were aware of future follow-ups and had more regular assessments (e.g. every 3 years). One Finnish cohort was examined irregularly at ages 5, 9, 16 and 30 years and achieved a retention rate of 54%; however, details on how they maintained contact with participants were not reported [11, 12]. Using phone and mail contact methods, a Canadian study was able to re-initiate contact with 29% of participants after a 20-year period of inactivity (last seen at age 15–17 years), a similar rate to the KYAMS (35%). The overall participation rate of this study (12.4%) was lower than the KYAMS [5]. Interestingly, this Canadian study had greater success in tracing men, whereas women were more likely to be contacted in the KYAMS [5]. A meta-analysis of studies of all ages found that predominantly women cohorts have better retention rates than those with more men, suggesting women are more likely to participate in ongoing follow-ups [12, 14].

We found that those who engaged in the 2005 follow-up were more likely to participate in the KYAMS compared to those not involved in the 2005 follow-up. Interestingly, the effect of participating in the 2005 follow-up on likelihood of KYAMS participation was substantially reduced when the analysis was restricted to only those who had been contacted. This indicates that the relationship between KYAMS participation and 2005 follow-up participation was largely mediated by the higher likelihood of being able to contact participants who attended the 2005 follow-up. Additionally, most participants of the 2005 follow-up had a mobile telephone number, which was independently associated with increased likelihood of being contacted but was not associated with increased likelihood of participating in the KYAMS.

Although having a mobile or landline telephone number was associated with greater likelihood of being contacted, we found that once contact had been achieved, contact by invitation letter was most likely to result in KYAMS participation. There could be several reasons for this. First, receiving a letter may have been a less intrusive contact method and allowed participants more time and flexibility to understand the study and its requirements. Second, we attempted to make contact with most participants by letter in the first instance; therefore the most motivated participants, who may have responded to *any* contact method, would have been most likely to respond to the first contact (i.e. letter). However, despite being sent later in the recruitment period, the second round of letters was nearly as effective as the first in translating KYAMS contact to KYAMS participation, casting doubt on this explanation. Third, participants not interested in participating may have been less likely to return the reply-paid envelope. These participants would be incorrectly classified as not contacted and would introduce an ascertainment bias, artificially inflating the apparent effectiveness of letter invitations in increasing likelihood of KYAMS participation.

The participant being the first point of contact was strongly associated with KYAMS participation. In many instances, we had no control over whether the participant or a family member was the first point of contact. Contact data from the Kidskin Study were first collected when the participants were still in school and likely residing in the family home. Most participants had moved out of the family home by the time of the KYAMS. Using Facebook did result in some extra instances of KYAMS contact and participation, but overall the success was limited, with only 15 (5%) of KYAMS participants contacted by Facebook.

Participants in the KYAMS were significantly more likely to be female, assigned to a Kidskin Study intervention group and have a parent with a tertiary education, than participants of the Kidskin Study baseline. Furthermore, compared to those who didn’t participate, KYAMS participants had lower parent-reported sun exposure in the previous holidays at the 1997, 1999 and 2001 Kidskin Study follow-ups. There was evidence that these findings were confounded by other variables such as 2005 follow-up participation. In multivariable logistic regression, parental education was not associated with either increased likelihood of being contacted for or participating in the KYAMS, and female sex and being assigned to an intervention group were associated with increased likelihood of KYAMS contact but were not significantly associated with KYAMS participation once contact had been made.

Many studies have reported on retention rates and strategies in longitudinal cohort studies. A recent systematic review and meta-analysis concluded that having a higher number of follow-up or reminder strategies was actually associated with reduced retention, [14] although perhaps this arises when studies struggling to retain participants resort to using other follow-up strategies. The review did, however, conclude that the use of more emerging follow-up strategies (text message, social media) were associated with improved retention [14].

Studies have consistently shown that incentives increase retention rates [14–16]. Invitation letters and phone calls are common methods for contacting cohort members [14]. Other cohort studies and randomised controlled trials have identified a key role for mobile phones in retention [17, 18] and some contemporary cohort studies use Facebook to maintain contact with or improve retention of participants [19, 20]. Past studies specifically addressing cohort retention after long periods of inactivity have concluded that the internet provides a useful tool for tracing participants through address and phone number details available online, but these studies were conducted in the mid-2000s and have not evaluated the use of social media [5, 6].

We faced some unique challenges when re-engaging the Kidskin Study cohort. First, contact information for most participants was last updated before the widespread use of mobile phones and the internet to communicate; therefore, we had few mobile phone numbers and no email addresses. Second, about one-third of the cohort had not been contacted for at least 10 years and the remaining two-thirds had not been contacted for 14 years or more. Third, most participants had moved out of the family home in the interval between the KYAMS and the previous follow-up; thus many were no longer residing locally or their contact information was out-of-date.

From our experience of the KYAMS we have learned that while advancements in technology have provided new communication methods, many participants still prefer to receive an invitation letter and indeed most KYAMS participants were contacted this way. Additionally, while mobile phones may make it easier to contact individuals, this may not be their preferred contact method. Participants we called were often busy and preferred to communicate by email or text message.

This analysis has limitations. It is retrospective; consequently, data were collected only partially systematically. For example, the number of letters sent was recorded but not the number of phone calls made. We therefore considered each phone number to be one contact attempt, which underestimates the number of phone calls made. Additionally, due to the higher rate of successful contacts, those who participated in the 2005 follow-up or who had a mobile phone number may have received more contact attempts. We also assumed that only those who responded to contact attempts had been contacted, but it is likely that some participants received letters or Facebook message requests and ignored them, leading to incorrect estimates of the number of participants contacted. Finally, the KYAMS participants were not representative of the Kidskin Study cohort and participants with or without an eye condition, such as myopia, may have been more or less likely to participate in the KYAMS. However, the non-representativeness of the sample would only impact the results of our analysis if the factor that made an individual more or less likely to participate (e.g. sex) was also associated with the contact method. For example, it is possible that, compared to men, females were more predisposed to participating in the KYAMS after receiving a letter, but it seems unlikely that contact method is associated with myopia status. Similarly, participation in the 2005 follow-up was associated with both an individual’s likelihood of participating and with contact method (e.g. mobile phone) but we were able to adjust for this, as well as sex, in the multivariable analysis.

## Conclusions

Re-engaging a cohort after a period of inactivity is a challenge, and changes in communication methods over time can lead to past contact information becoming out-dated. After at least a 10-year period of inactivity, 599 (35.4%) of eligible Kidskin Study participants or their family members were contacted about the KYAMS and 303 (17.9%) original Kidskin Study cohort members participated in the study. Most participants were contacted by letter, but up-to-date mobile phone numbers can improve likelihood of making contact. Once contact was established, direct contact with the participants themselves (i.e. not family members) and the use of invitation letters were most effective at recruiting participants.

## Data Availability

The datasets analysed during this study are not publicly available to protect the privacy of the participants. Non-identifying data are available on from the corresponding author on reasonable request.

## Abbreviation

KYAMS: Kidskin young adult myopia study
